# Bidirectional Association between Lichen Planus and Hepatitis C—An Update Systematic Review and Meta-Analysis

**DOI:** 10.3390/jcm12185777

**Published:** 2023-09-05

**Authors:** María García-Pola, Lucia Rodríguez-Fonseca, Carlota Suárez-Fernández, Raquel Sanjuán-Pardavila, Juan Seoane-Romero, Samuel Rodríguez-López

**Affiliations:** 1 Department of Surgery and Medical-Surgical Specialties, Faculty of Medicine and Health Sciences, University of Oviedo, 33004 Oviedo, Spain; uo166116@uniovi.es (L.R.-F.); uo245187@uniovi.es (C.S.-F.); raquelsanjuann@hotmail.com (R.S.-P.); uo238400@uniovi.es (S.R.-L.); 2 Department of Surgery and Medical-Surgical Specialties, School of Medicine and Dentistry, University of Santiago de Compostela, 15780 Santiago de Compostela, Spain; jseoaner@hotmail.com

**Keywords:** lichen planus, oral lichen planus, hepatitis C, systematic review, meta-analysis, skin diseases, epidemiology, comorbidity

## Abstract

Lichen planus (LP) is a chronic, inflammatory mucocutaneous disorder associated with systemic diseases such as hepatitis C (HCV). The objective of this study is to evaluate the association between LP and HCV bidirectionally through a systematic review and meta-analysis. A comprehensive search of studies published was performed in the databases of PubMed, Embase, and Web of Science. Out of 18,491 articles, 192 studies were included. The global prevalence of HCV positive (HCV+) in LP patients registered from 143 studies was 9.42% [95% confidence interval (CI), 7.27–11.58%], and from these, 84 studies showed HCV+ 4-fold more frequent in LP than a control group (OR, 4.48; 95% CI, 3.48–5.77). The global prevalence of LP in patients HCV+ recorded from 49 studies was 7.05% (95% CI, 4.85–9.26%), and from these, 15 registered a 3-fold more LP in HCV (OR, 3.65; 95% CI, 2.14–6.24). HCV+ in LP patients showed great geographic variability (OR, 2.7 to 8.57), and the predominantly cutaneous location was higher (OR, 5.95) than the oral location (OR, 3.49). LP in HCV+ patients was more frequent in the Eastern Mediterranean (OR, 5.51; 95% CI, 1.40–15.57). There is a higher prevalence of HCV+ in LP and vice versa than in the control group, especially in certain geographical areas that should be taken into consideration when doing screening in countries with an upper prevalence of HCV among the general population.

## 1. Introduction

Lichen planus (LP) is a chronic relapsing inflammatory mucocutaneus disease mainly involving the skin, oral, and genital mucosa and appendages such as nails and hair [1]. Estimations on the prevalence of LP are between 0.14 and 1.27% of the general population [2]. Although its etiopathogenesis remains unclear, LP is attributed to T-cell-mediated inflammatory disease processes that result in apoptosis of the basal keratinocyte layer of the epithelium [3]. The relationship between hepatitis C virus (HCV) infection and lichen planus was first described in 1991, a year after the discovery of the virus itself [4].

The estimated global HCV prevalence in 2015 was 1.0% (95% uncertainty interval, 0.8–1.1) [5], correlating to 71.1 million individuals infected with HCV and taking into account that only 20% of individuals with HCV know their diagnosis [6]. In 2016, the World Health Organization (WHO) adopted a global hepatitis strategy to eliminate viral hepatitis as a public health threat by 2030, with an 80% reduction in incidence cases of HCV and a 65% reduction in mortality [7].

Previous systematic reviews and meta-analyses examined the association between LP and HCV with odd ratio (OR) variation between 2.8 [8] and 7.08 [9] probably due to the design of the study, the location of LP, the year of study, and the geographical area included [10,11,12,13,14,15]. Otherwise, it seems that among HCV-infected patients, the prevalence of oral lichen planus (OLP) is lower than vice versa [13,16].

In order to determine whether it is advisable to carry out screening in patients with LP for hepatitis C and determine the expression of LP as an extrahepatic manifestation of hepatitis, we performed an updated systematic review and meta-analysis of the published literature with the following objectives: (1) to determine the prevalence and the association of hepatitis C in individuals with lichen planus; (2) to determine the prevalence and the association of lichen planus in individuals with hepatitis C; and (3) to analyze the association between prevalence with possible related variables and the need to perform hepatitis C screening among patients with OLP and vice versa.

## 2. Materials and Methods

This review was conducted on the basis of the Preferred Reporting Items for Systematic Reviews and Meta-analyses (PRISMA) guidelines (Supplementary Table S1. PRISMA item checklist) [17]. The protocol was registered in the PROSPERO database (ID: CRD42020153380). The approval of the ethics committee was not applied.

### 2.1. Search Strategy

Three authors independently (G-P, R-F, and R-L) searched the databases PubMed, EMBASE, and Web of Science (all collections) until 30 June 2023. The search was limited to human studies in the English language. Additional literature was sought through reference lists of the included articles. The combined search terms lichen planus (OR cutaneous lichen planus OR oral lichen planus OR lichen planopilaris OR genital lichen) AND hepatitis C (OR hepatitis OR liver disease) were used. For search terms, see Supplementary Appendix S1.

### 2.2. Selection Process

Studies were selected according to the following inclusion criteria: (1) observational studies examining the association between lichen planus and hepatitis C, including cohort studies ≥ 15 cases, cross-sectional and case-control studies, prospective or retrospective. The eligibility criteria for observational studies were based on CoCoPop mnemonic (condition, context, and population) [18], and for case control on PICOS strategy (participants, interventions, comparators, outcomes, and study design) [19]; (2) participants of all age groups and both genders; (3) reports of prevalence or incidence of LP in hepatitis C, and vice versa, or presence of sufficient information to estimate the prevalence and crude OR; (4) LP disease diagnosed and evaluated clinically or histologically, or through medical database, survey, or questionnaire; (5) HVC diagnosed and evaluated by laboratory testing anti-HCV antibodies or PCR-screening, or through medical database, survey, or questionnaire.

Exclusion criteria were as follows: (1) not LP, such as lichenoid reaction [2,20] or not hepatitis C patients, and (2) from the literature, duplicate publications, conferences, case reports fewer than 15 screening for viral C hepatitis, reviews, unpublished studies, editorials, and non-research letters.

### 2.3. Data Collection Process

Disagreements were resolved by discussion and consensus with a fourth reviewer (S-F). The following data were extracted and recorded from each eligible study: (1) first author; (2) year of publication; (3) city and country where the fieldwork is carried out; (4) study design; (5) number of case-patients (female/male); (6) age of case-patients (mean ± standard deviation and range); (7) number of control individuals (female/male); (8) age of control individuals (mean ± standard deviation and range); (9) location of LP; (10) diagnosis tool of LP; (11) diagnosis tool of HCV; (12) outcomes; and (13) screening recommendation for hepatitis C. When a single study was described by more than one publication, we included the most consistent, or most recent, report.

### 2.4. Quality Assessment

For each included study, three independent reviewers (G-P, R-F, and R-L) used the Newcastle-Ottawa Scale (NOS) to evaluate the quality of case-control studies [21], and for cohort studies, the guide of the Joanna Briggs Institute (JBI) [22]. A discussion with a fourth reviewer (S-F) was conducted to resolve any disagreements in the quality assessment. NOS included eight domains; every single question received 1 point (marked as a star *), except for the comparability item, which could be awarded with 2 points. The JBI scale also included nine questions. Therefore, in both scales the maximum total score is 9 points. In both tools, scores of 7 to 9 are considered high quality (low risk of bias), scores of 4 to 6 are medium, and scores of <4 are low quality (high risk of bias).

Two authors (G-P and R-F) used the Review Manager system (RevMan, version 5.3.5; The Nordic Cochrane Centre, Copenhagen, Denmark) to calculate the pooled OR and the consistency. The Q-value (chi square) and the I^2^ statistics were used to evaluate heterogeneity. The following cut-offs were used for reporting heterogeneity: <25% non-heterogeneity, 25–49% low heterogeneity, 50–74% moderate heterogeneity, and ≥75% high heterogeneity [23]. Initially, the fixed-effects model was used, and if the heterogeneity was statistically significant (*p* < 0.05), a random-effects model was applied with the Mantel–Haenszel method. In addition, to assess the effect of each study and publication bias, three authors (S-F, S-P, SR) used a funnel plot and Egger’s statistical regression test (Epidat version 3.1).

### 2.5. Statistical Analysis

Subgroups included the following: diagnosis methods of LP and hepatitis viral C; location of LP [predominantly cutaneous with mixed variant cutaneous and mucous (CLP), oral (OLP), and planopilaris lichen (PPL)]; sample size (<100; ≥100); study design by time period (prospective, retrospective, ambispective); control group (matched and type); risk of bias by study quality according to the Newcastle-Ottawa Scale; publication decade; and geographic area. For the global computation of the OR, the number of patients with the highest titers of antibodies or PCR positivity was chosen, and in the case of presenting two control groups, the most representative was selected (S-F, S-P, and S-R). Geographical areas were considered according to the distribution of the World Health Organization [24].

Analysis with SPSS software (version 27) was used to calculate pooled proportions with 95% confidence intervals (CIs) for the overall populations and for all subgroups.

## 3. Results

### 3.1. Search Results and Description of the Included Studies

**Figure 1:**
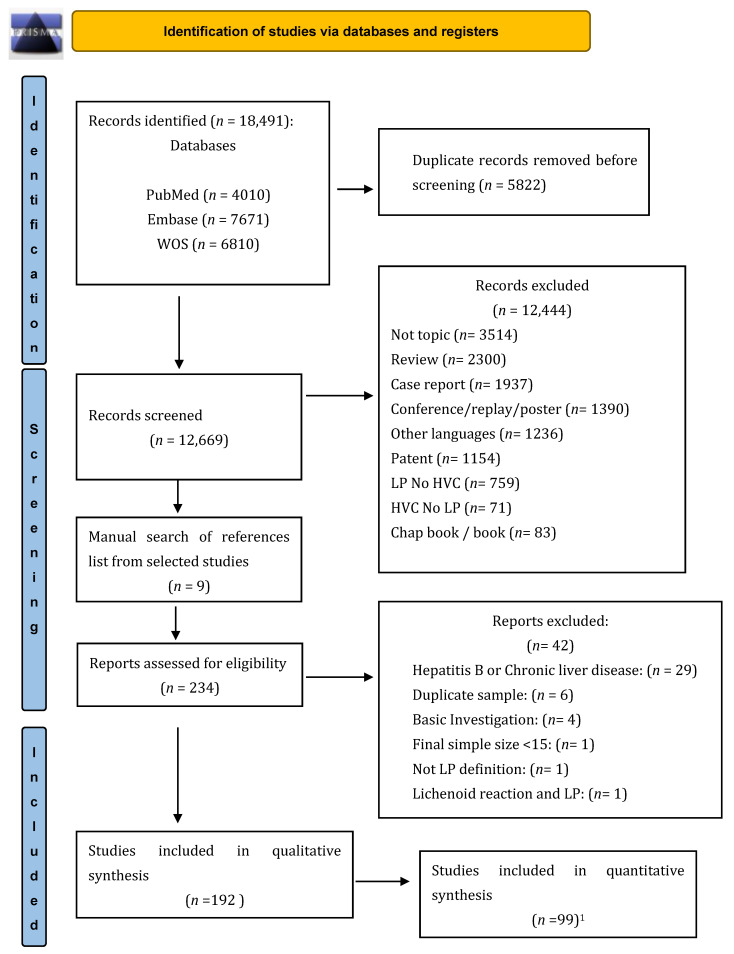
Flow diagram with the information through the phases of study selection based on PRISMA guideline [17]. ^1^ LP: lichen planus; HCV: hepatitis C virus.

Literature yielded a total of 18,491 references, and once duplicates were excluded (*n* = 5822), 234 studies were full-text reviewed. In total, 192 studies were included in the systematic review, of which 99 studies were included in the quantitative analysis, shown in Figure 1 [8,10,25,26,27,28,29,30,31,32,33,34,35,36,37,38,39,40,41,42,43,44,45,46,47,48,49,50,51,52,53,54,55,56,57,58,59,60,61,62,63,64,65,66,67,68,69,70,71,72,73,74,75,76,77,78,79,80,81,82,83,84,85,86,87,88,89,90,91,92,93,94,95,96,97,98,99,100,101,102,103,104,105,106,107,108,109,110,111,112,113,114,115,116,117,118,119].

### 3.2. Prevalence and Association of Hepatitis C in Patients with LP

From 143 studies, the overall pooled prevalence of HCV in patients with LP was 9.42% (95% CI, 7.27–11.58%). From them, sixty cohorts of 59 studies reported were only included for qualitative study, and the overall pooled prevalence of HCV in LP patients was 8.31% (95% CI, 5.53–11.10%) [120,121,122,123,124,125,126,127,128,129,130,131,132,133,134,135,136,137,138,139,140,141,142,143,144,145,146,147,148,149,150,151,152,153,154,155,156,157,158,159,160,161,162,163,164,165,166,167,168,169,170,171,172,173,174,175,176,177,178]. Among 30 children, none were HCV antibody-positive [161]. When considering studies that analyze genital LP (there were none in females (0/120) [128,136] and only one in males (1/150) [121,170]. When considering studies with only lichen planopilaris, the prevalence was 0.87% (HCV 3/342) [134,156]. Detailed information on the sample to the included studies is provided in Supplementary Table S2 [8,10,25,26,27,28,29,30,31,32,33,34,35,36,37,38,39,40,41,42,43,44,45,46,47,48,49,50,51,52,53,54,55,56,57,58,59,60,61,62,63,64,65,66,67,68,69,70,71,72,73,74,75,76,77,78,79,80,81,82,83,84,85,86,87,88,89,90,91,92,93,94,95,96,97,98,99,100,101,102,103,104,105,106,107,108,109,110,111,112,113,114,115,116,117,118,119,120,121,122,123,124,125,126,127,128,129,130,131,132,133,134,135,136,137,138,139,140,141,142,143,144,145,146,147,148,149,150,151,152,153,154,155,156,157,158,159,160,161,162,163,164,165,166,167,168,169,170,171,172,173,174,175,176,177,178,179,180,181,182,183,184,185,186,187,188,189,190,191,192,193,194,195,196,197,198,199,200,201,202,203,204,205,206].

The total number of studies for meta-analysis for determining HCV in LP was 84 and included 13,495 LP patients who were 969 HCV+, and 401,386 individuals served as a control group, with 1515 HCV+ subjects. The pooled OR acquired was OR 4.48 (95% CI, 3.48–5.77). The OR of HCV seropositivity in patients with LP varied between 0.23 (95% CI, 0.01–5.85) [113] and 67.67 (95% CI, 7.95–575.68) [29]. The heterogeneity was moderate, with an I^2^ of 60%. The results of this section are reflected in Table 1 and Supplementary Table S3. In seventeen studies, no seropositive patients were found in either group. The proportion of HCV+ subjects was higher in the LP group compared with controls, except for seven studies [41,47,92,97,103,113,118]. The sensitivity analysis determined that the funnel plot would be more symmetrical if five studies were excluded [29,37,47,59,118] and was corroborated by the Egger test.

### 3.3. Prevalence and Association of LP in Patients with Hepatitis

From forty-nine studies, the overall pooled prevalence of LP in HCV patients was 7.05% (95% CI, 4.85–9.26%). From these, in thirty-four cohorts, the overall pooled prevalence of LP was 7.75 (95% CI 4.84–10.66%) in patients with HCV [179,180,181,182,183,184,185,186,187,188,189,190,191,192,193,194,195,196,197,198,199,200,201,202,203,204,205,206]. Detailed information on the sample to the included studies is provided in Supplemental Table S2.

The other fifteen studies met the inclusion criteria for meta-analysis. The total number of patients with HCV+ in the included studies was 59,221 and with LP was 214, and 233,335 individuals served as a control group, with LP+ 231 used to analyze the association between hepatitis and LP. The OR registered was OR 3.65 (95% CI, 2.14–6.24). The OR of LP in patients with HCV+ varied between 0.11 [95% CI, 0.01–2.03] [83] and 12.02 [95% CI, 6.56–22.01] [80]. The results of this section are reflected in Table 2 and Supplementary Table S4. The proportion of HCV+ subjects was higher in the LP group compared with controls, except for one study [83]. The heterogeneity was I^2^ 61%. The sensitivity analysis determined that the funnel plot would be more symmetrical if one study was excluded [79], which was corroborated by the Egger test.

### 3.4. Subgroup Analyses of Association of Hepatitis C in Patients with LP

When the subgroup analysis was made by collecting studies of geographic areas, the highest OR was found in studies from the African region (OR 8.57; 95% CI, 1.54–47.76) and from Southeast Asia (OR 7.73; 95% CI: 2.85–20.92). When considering countries, the variation was higher, with Iraq and Egypt being close ten-fold in Thailand and Japan (Table 3). Within the same country, in Italy, there were also variations between Campania (OR 1.91; 95% CI, 1.43–2.57) [37] and Brescia (OR 10.59; 95% CI, 3.00–37.35) [10]. In the same region of Piemonte, north-west of Italy, in 1996, OR was higher (OR 8.32; 95% CI, 2.33–29.66) [38] than in 2017 (OR 3.21; 95% CI, 0.68–15.26) [30] (Supplementary Table S5).

With regard to the difference in decades of publications, in the last publication, from 2011, the OR was lower (OR 3.84; 95% CI, 2.51–7.32) than in the decade between 1991 and 2000 (OR 5.17; 95% CI, 3.66–7.32).

A total of 60 out of 84 studies in the quantitative analysis had an NOS score of 7 or higher (OR 3.98; 95% CI, 3.05–5.20). The association remained significant when the analysis was restricted to studies where LP was clinical and biopsy diagnosed (OR 4.19; 95% CI, 3.19–5.50), and when the diagnosis of HCV was performed with antibodies (OR 4.17; 95% CI, 2.75–6.33), or by PCR and antibodies (OR 5.11; 95% CI, 3.94–6.64). In 12 studies, there was a higher number of positives for antibodies than PCR [34,37,38,54,63,67,70,72,81,89,100,105], compared with seven studies where there were no differences [55,57,92,98,99,106].

According to the control group, when the control group came from the general population, the association of HCV in patients with LP was lower (OR 2.68; 95% CI, 2.15–3.34), than when the control group was healthy subjects (OR 6.26; 95% CI, 3.77–10.40). When compared with psoriasis, it was somewhat lower (OR 3.64; 95% CI, 1.35–9.86) than when compared with other skin diseases or other oral diseases (OR 4.73; 95% CI, 3.64–6.14).

The risk of HCV in LP-matched gender with the control group was very similar (OR, 4.43; 95% CI, 3.02–6.48) to the matched age (OR, 4.29; 95% CI, 2.89–6.36). Other similar higher data was observed in the prospective studies (OR 4.53; 95% CI, 3.46–5.92) compared with the retrospective ones (OR 3.44; 95% CI, 2.01–5.87). When the sample size was greater than 100 subjects, the OR was lower (OR 4.18; 95% CI, 2.84–6.14).

The risk in patients with cutaneous and variant LP (with or without mucous location) was higher (OR 5.95; 95% CI, 4.09–8.66) than that observed in patients with predominantly or only oral location (OR 3.49; 95% CI, 2.00–4.87) [8,10,25,26,27,30,32,36,37,38,40,41,47,50,51,52,55,57,61,68,69,72,73,75,76,77,78,79,81,84,89,92,93,94,95,96,98,101,102,103,109,110,117,118].

The authors of twenty-two studies from 84 have declared their support for the carrying out of antibody screening for hepatitis C [10,34,39,48,55,58,62,73,77,81,89,117], especially in the following situations: erosive LP [38,60,102,105] patients with different types of LP [29], risk factors for hepatitis infection [33], areas of high HCV prevalence [8], high-level liver enzymes [52,65] or to at least make the patient aware of the possible association between LP and HCV [96], while others do not [75,85,97,98,103,106,113,119]. Another opinion was that in patients with risk factors for chronic liver diseases or in patients with risk factors for hepatitis C, the chart should be taken with care [35].

### 3.5. Subgroup Analyses of Association of Patients with LP in Hepatitis C Patients

When the subgroup analysis was made by collecting studies from geographic areas according to WHO regions, the Americas, the Eastern Mediterranean, and the Western Pacific region, the risk was two-fold higher (OR 2.42, OR 5.51, and OR 4.79, respectively). There was no association in European regions (OR 2.08 [0.95–4.52]) (Table 4).

A total of ten studies in the quantitative analysis had an NOS score of 7 or higher (OR, 4.16; 95% CI, 2.11–8.23). The association remained significant when the analysis was restricted to studies where LP was diagnosed by laboratory (OR, 3.03; 95% CI, 1.94–4.74) or by records (OR, 5.18; 95% CI, 1.04–25.80).

In regard to the difference in decades of publications, in the most recent study, the OR was higher (OR, 12.02; 95% CI, 6.56–22.01) [79]. The difference obtained according to the control group also stands out as the OR when compared with healthy subjects (OR, 2.97; 95% CI, 1.63–5.44). The association remains stable when studies are divided by matched gender and age (OR, 3.41; 95% CI, 1.28–9.08).

When the sample size was greater than 100 subjects, the OR was higher (OR 4.84; 95% CI, 1.98–11.78). In the retrospective studies, the OR was higher (OR, 5.18; 95% CI, 1.04–26.50) compared with the prospective ones (OR, 3.03; 95% CI, 1.94–4.74).

## 4. Discussion

The coexistence of LP with HCV has been known for three decades, but the topic is still controversial. LP is a chronic inflammatory mucocutaneous condition, and HCV is one of the major causes of chronic liver disease. Extrahepatic manifestations of HCV infection can affect a variety of organ systems with significant morbidity and even dermatological disorders such as LP.

In this update, we include studies published in the English language since the LP and hepatitis C associations were recognized. The results of the present meta-analysis have revealed that LP patients have a four-fold higher risk for HCV infection (OR, 4.48; 95% CI, 3.48–5.77) and suggest a firm link between LP and HCV infection. In the same way, we have three times corroborated the presence of LP in HVC patients (OR, 3.65; 95% CI, 2.14–6.24).

Our results draw near to those provided by Shengyuan et al. [13], in 2009, by Lodi et al., in 2010 [10], and recently by González-Moles et al. [15]. We reduced the chance of systematic error or bias by considering different methodological aspects that increase the validity of the study, highlighting different variables of diagnostics for LP and HCV, the control group, sample size, and geographic regions.

Age and sex are relevant potential confounding factors. LP affects with a predilection for a mean age from the fourth decade [1], and most new HCV infections occurred in those aged 20–39 years [207,208]. The results of our meta-analysis from 41 of the 84 included studies of case-control design; with the control group matched by sex and age, the OR remains at 3.85 [95% CI, 2.66–5.56 (I^2^:51%)].

To reduce the risk of bias provided by the LP diagnostic method, we excluded no LP, such as lichenoid reactions, or when the origin or definition of the LP was not stated. In addition, all the subgroups considered, whether through clinical and/or histopathological diagnosis or through the charts, maintained an OR greater than 3.62. In the same way, considering the different diagnostic methods for HCV, the OR was always higher than three-fold, being higher with PCR-RNA and antibodies than with only antibodies (OR, 5.11 vs. OR, 4.24). These results should be interpreted with care, as the association with LP is greater when hepatitis is in the acute phase. HCV RNA becomes reliably detectable within 2–3 weeks after viral exposure, and HCV antibody seroconversion among immunocompetent persons occurs 2–3 months after exposure [207].

The selection of control groups to contrast with the cases is fundamental for the establishment of an association between them. We assessed studies comparing the prevalence of HCV in those with LP in various control types separately because, depending on control types, we addressed different questions, finding in our results a great variability in the OR. In the opinion of Shengyuan et al. [13], healthy populations and blood donors may not represent appropriate control groups because they may have a lower seroprevalence. Chainani et al. [209] express their view that patients attending a tertiary referral center differ from the general population. In fact, in these three groups of populations, we found an OR of 6.26, 6.69, and 2.68, respectively. The results were more similar when compared with other dermatosis (38 studies, OR 4.73, I^2^: 27%) or concretely with psoriasis (3 studies; OR 3.64, I^2^: 0%) although it is understood that they are not representative of the entire population. It is probable that the OR differs from healthy individuals in a series of factors that are related to the disease process in general and that could be directly or indirectly related to exposure [210].

Thirty-four out of 84 studies included more than 100 subjects, and the OR was lower when there were fewer than 100 patients (OR, 4.18 vs. OR, 4.89). Paradoxically, when the design was prospective, the OR was higher than when it was retrospective (OR, 4.53 vs. OR, 3.44).

Our study further supports the notion that the relationship between these two diseases is connected to the geographic site not only within the same continent or sub-regions but also within the same country. Previously, the higher prevalence of HCV patients with LP was commonly seen in Japan [13], the Mediterranean [10,13], the Middle East, and Asia [11]. We confirmed a higher prevalence in Japan and Thailand, as well as in Iraq, Egypt, and Nigeria. The association in most regions analyzed presented an OR higher than 2.87, but given the OR and prevalence, with our figures, it would be interesting to clarify several aspects. When we considered the prevalence of HCV in patients with LP by WHO regions, we found it to be lower in South-East Asia (3.43%) and higher in America (11.54%), Africa, and the Western Pacific (both upper than 17%). However, figures of prevalence and the OR contrast between continents and countries. For example, in European countries such as the Netherlands [75], the United Kingdom [68], Poland [119], and Serbia [36], no association was recorded, although it should be considered that the sample in these last two studies was 84 and 48 patients, respectively. In South-East Asia, India (OR, 6.70) contrasted with Nepal (zero patients with HCV in the control group and case group) and the Western Pacific, where in China the estimated OR was 0.69 and in Japan the OR was 9.75. Furthermore, these studies should consider its heterogeneity in part in terms of design variety or sample size.

The prevalence of HCV infection in general populations also varies by geographic region. Of the countries cited in this study, the country with the lowest prevalence of HCV antibodies in the general population is the Netherlands (0.1%), and the country with the highest seropositivity is Egypt (6.3%) [211]. The preventive action of some countries on the transmission of HCV has been decisive in reducing its prevalence among the general population. Even so, it is difficult to justify that, in the same way, a decrease in HCV has been observed in patients with LP in recent years in countries such as the US, Germany, or Italy. Thus, the prevalence of HCV+ in LP in the region of Turin between March 1992 and July 1994 was 27.1% [38], and between January 2015 and May 2017 it was 2.6% [30]. Comparatively, among the general population prior to 2010, hepatitis C figures of 5.2% were recorded in the Italian adult population, with a north-south gradient [212], and have progressively decreased to 2% [213], being in the year 2015 1.1% (0.7–2.7) [5]. Another country to highlight in our analysis is India, due to the fact that the OR has increased in the last decade, OR 9.04, and does not correspond to the prevalence of HCV in the general population of 0.5–1.5% [5].

This geographical heterogeneity is arduous to clarify and could be explained by the intervention of immunogenetic factors, such as different genotypes in hepatitis C and different human leukocyte antigens (HLA) in LP.

The limited number of studies that analyze the genotypes associated with LP makes it difficult to interpret their role in the prevalence of LP. Among the genotypes observed in patients with LP and hepatitis C, 1a and 1b predominated [68,214,215], which were the most frequently observed in Europe in 1999 [216], since currently in the UK genotype 3 predominates (43.8%) [5]. The study carried out in India by Khaja et al. [217] found that almost 70% of patients with LP had genotype 1b, while in donors it was 34.1%. A recent study revealed that the most prevalent genotype subtypes in Southeast Asia were 1b (26.3%) and 1a (21.3%). In addition, among the general population, the presence of the type 3 genotype has increased in Brazil (30%) [5], the UK (43.8%) [5], Pakistan (79.9%) [5], and Thailand (45.8–47.8%) [5,218], and the type 4 genotype in Egypt (90%) [5]. However, the presence of these genotypes in patients with LP has not been verified.

From the HLA point of view, DR typing of major histocompatibility complex class II alleles may influence the development of OLP in patients with HCV infection. HLA-DR6 allele is more frequent in Italian HCV+ and exclusive oral LP patients than in Italian and British OLP patients without HCV infection (51.6% vs. 17.7% vs. 16.7%), but in this study, HLA-DR6 was not analyzed among British patients with OLP and HCV [219]. In the Japanese population, Nagao et al. identified single-nucleotide polymorphisms in the HLA-DR/DQ, in *NRP2* and *IGFBP4* loci, which increase and reduce the risk of LP, respectively, and those authors propose that these genetic variants might be used to identify patients with HCV infection who are at risk for lichen planus [220].

The association of HCV infection with LP is difficult to justify taking into account the geographical differences, such as the strong association from the Eastern Mediterranean (OR 5.51; CI, 1.40–15.57) and the lack of association in the European Region (OR 1.47; CI, 0.79–2.73). This association could occur because of the ability of HCV to replicate in the skin and oral mucosa. Although HCV replicates it and is observed mainly in hepatocytes, some investigators have identified viral RNA in the cutaneous [221,222,223] and oral mucosa [224] of patients with chronic hepatitis C, regardless of whether or not they have LP lesions. However, other studies did not find HCV RNA in the cutaneous [225] and oral mucosa samples of patients with OLP and HCV infection [226]. Therefore, evidence to support the epithelial tropism of HCV is insufficient [227].

In relation to the location of the LP, HCV has not been present in patients with predominantly genital locations and in a proportion of 0.87% in patients with lichen planopilaris, so we should not draw a conclusion, especially considering that there was only one case-control study. However, the meta-analysis shows that the OR of HCV in LP with a greater predominance of cutaneous and varied involvement is greater than the oral predominance (OR, 5.95 vs. OR, 3.49). This contrasts with the results of Shengyuan et al. [13], who observed that the cutaneous type was statistically insignificant (OR, 10.2 [95% CI, 0.4–273.5]) versus the isolated mucosal type of LP (OR, 4.8 [95% CI, 3.0–7.7]).

The manifestations of HCV infection in the skin and mucous membranes of patients with LP do not necessarily imply that it is due to its etiological association. In fact, HCV has lymphotropism, which is not only associated with specific T-cell responses but is also responsible for B-lymphocyte expansion. The consequence of the stimulation of B lymphocytes involves the production of auto-antibodies, potentially leading to a range of immunological alterations and B-lymphocyte proliferative disorders, such as diabetes mellitus and autoimmune thyroiditis, and those diseases are common in LP [228].

Another explanation could be justified by unbalanced oxidative stress. It has been proposed that there is an alteration of the balance between oxidant and antioxidant levels in the saliva and serum/plasma in patients with CLP [229]. In OLP [230], there are increasing levels of nitric oxide, malondialdehyde, and superoxide dismutase and decreasing levels of catalase. Furthermore, the cumulative oxidative burden is likely to promote both hepatic and extrahepatic conditions precipitated by HCV [231]. It has recently been proposed that symmetric dimethylarginine in serum could be a marker of oxidative stress among patients with LP and HCV [232]. On the other hand, the study by Khadem Ansari et al. showed that genotypes 1a and 1b are more associated with a higher level of oxidative stress, and we have already commented that these genotypes are frequent among patients with LP and HCV [233].

The question of screening patients with LP for HCV is also difficult to clarify from the analyzed studies. Different screening models have been proposed for hepatitis with cost-effective positive results. Lapane et al., in 1998, from the database National Hepatitis Surveillance Program, suggested testing for HCV if the probability of HCV was determined to be higher than 7% [234]. Using different modeling techniques to demonstrate that one-time universal screening for HCV in adults aged ≥ 18 years is cost-effective compared with birth-cohort screening from 1945 through 1965. For these reasons, a one-time, routine, opt-out HCV test for all individuals aged 18 is recommended [207,235]. The goal to eliminate viral hepatitis by 2030 has generated the concept of so-called microelimination of HCV infection in target groups, medical conditions, specific subpopulations, or entire countries [236]. The analysis that we present would be a starting point to consider LP as a medical condition for which screening should be performed following the concept proposed by some authors, where the prevalence of HCV is high [237].

The main limitations of the study are related to those derived from the exclusive selection of articles in English, as well as the difference in sample sizes that did not allow the analysis of some subgroups and the heterogeneity of some results. On the other hand, the severity and chronicity of the HCV infection could be another risk of bias.

## 5. Conclusions

In conclusion, even though the prevalence of HCV has decreased among LP patients and in spite of the improvement of LP with antiviral treatment [238], our meta-analysis supported a statistically significant bidirectional association by epidemiological link. It has been evident that the association is higher in LP with cutaneous predominance than mucosal location, especially in certain geographical areas. Therefore, both points must be considered by health professionals to evaluate the spread of hepatitis C in geographical areas with a major expression of hepatitis C and new investigations in search of the genetic association of both pathologies should be carried out.

## Figures and Tables

**Table 1:** Pooled OR and 95% Confidence Interval (CI) of Hepatitis C (HCV) in Lichen planus (LP). ^1^. Cohort from Campania [35]. ^2^ Cohort from Sicily [38]. ^3^ Geographical location by WHO. https://www.who.int/countries (accessed on 30 June 2023) [24]. ^4^ Two studies used 2 control groups [37,43]. NA: not applicable. ^5^ Same control group for cutaneous and for OLP.

	Number of Studies	OR (95% CI)	I^2^	Q Test Chi^2^	Tau^2^	HCV+ in LP (n)	HCV+ in Control Group
All studies	84 (87cohortes)	4.48 [3.48–5.77]	60%	165.42	0.47	969+/13,495	1515+/401,386
Diagnosis LP							
Biopsy & Clinical	59	4.19 [3.19–5.50]	38%	71.34	0.23	668+/6336	788+/66,623
Clinical and/or Biopsy	15	6.37 [2.69–15.11]	82%	64.99	1.81	151+/2126	641+/324,659
Charts	10	3.62 [2.14–6.11]	57%	18.81	0.29	150+/5033	86+/10,104
Diagnosis HCV							
HCV antibodies ^1^	49	4.24 [2.83–6.35]	72%	119.61	0.85	571+/5071	1158+/378,090
HCV-RNA	2	16.33 [3.30–80.83]	0%	0.39		24+/55	1+/55
HCV antibodies + HCV-RNA ^2^	19	5.11 [3.94–664]	9%	18.70	-	209+/1765	153+/3741
Charts	7	3.21 [1.57–6.59]	78%	27.69	0.58	138+/5462	196+/18,821
Anamnesis	8	3.12 [1.44–6.75]	0%	1.43		27+/1232	7+/1279
Geographic location ^3^							
African Region	2	8.57 [1.54–47.76]	0%	0.01	-	18+/99	1+/54
Americas Region	7	4.71 [2.26–9.82]	48%	11.47	0.43	42+/669	341+/46,536
Eastern Mediterranean	18	5.95 [2.50–14.40]	74%	53.51	1.88	113+/1748	348+/323,154
European Region	34	3.78 [2.86–5.00]	27%	33.36	0.10	567+/6335	328+/14,621
South-East Asia	13	7.73 [2.85–20.92]	0%	0.98	-	31+/797	2+/774
Western Pacific	9	2.87 [1.48–5.56]	75%	30.93	0.64	151+/3574	475+/15,299
Multinational	1	9.65 [5.61–16.61]	NA			47+/273	20+/948
Publication date							
1991–2000	17	5.17 [3.66–7.32]	35%	21.49	-	211+/1246	52+/1365
2001–2010	35	5.04 (3.29–7.72)	72%	92.86	0.68	500+/3931	1227+/376,569
≥2011	32	3.84 (2.51–5.87)	55%	51.03	0.44	258+/8318	236+/23,452
Risk of bias							
Low	60	3.98 [3.05–5.20]	51%	95.84	0.30	814+/11,617	547+31,485
Medium	24	5.63 [3.10–10.23]	71%	61.08	1.07	155+/1878	968+/369,901
Matched							
Yes gender	35	4.43 [3.02–6.48]	49%	46.60	0.35	309+/7251	186+/17,545
Yes age	33	4.29 [2.89–6.36]	50%	45.69	0.36	292+/7106	183+/17,400
Yes gender & age	41	3.85 [2.66–5.56]	51%	59.78	0.41	322+/8834	318+/27,592
Not gender	32	4.74 [3.10–8.26]	72%	91.9	0.70	491+/3454	1177+/372,766
Not age	29	4.82 [3.04–7.66]	73%	89.38	0.75	429+/2987	1167+/372,384
Not dif gender	7	4.95 [2.59–9.47]	0%	3.11	-	50+/705	12+/695
Not dif age	12	7.34 [4.83–11.14]	0%	7.73	-	212+/1493	26+/1225
Control group ^4^							
Donors	9	6.69 [1.69–26.42]	82%	38.33	2.91	26+/592	400+/327,657
Not specified	2	9.65 [1.15–81.02]	-			8+/106	1+/85
Health	11	6.26 [3.77–10.40]	0%	4.72	-	97+/958	25+/938
Volunteers	5	5.21 [2.14–12.71]	0%	0.54	-	38+/283	5+/231
Same Department							
(Other pathology)	38	4.73 [3.64–6.14]	27%	39.98	0.57	330+/4109	406+/10,550
Records database	7	3.81 [1.96–7.41]	75%	16.06	0.39	131+/4580	71+/9267
Psoriasis	3	3.64 [1.35–9.86]	0%	0.00	-	16+/146	11+/160
General population	2	2.68 [2.15–3.34]	32%	1.48	-	244+/927	168+/1935
Other Department	8	2.65 [1.63–3.34]	42%	8.66	-	41+/837	46+/1730
Sample size							
<100	50	4.89 [3.88–6.18]	28%	4599	-	290+/2740	780+/57,728
≥100	34	4.18 [2.84–6.14]	74%	112.90	0.62	679+/10,755	735+/343,658
Time period							
Prospective	66	4.53 [3.46–5.92]	32%	73.86	0.26	482+/5989	810+/60,267
Retrospective	15	3.44 [2.01–5.87]	66%	35.49	0.48	236+/6433	218+/19,809
Ambispective	3	8.55 [1.21–60.36]	96%	55.86	2.84	251+/1073	487+/321,310
Location ^5^							
Predominant cutaneous	40	5.95 [4.09–8.66]	52%	68.87	0.51	326+/6851	740+/377,516
Predominant oral	44	3.49 [2.00–4.87]	62%	81.31	0.41	642+/6436	775+/23,701
Lichen Planopilaris	1	2.10 [0.09–51.86]				1+/208	0+/208

**Table 2:** Pooled OR and 95% Confidence Interval (CI) of Lichen planus in Hepatitis C patients. ^1^ Geographical location by WHO. https://www.who.int/countries. https://www.who.int/countries (accessed on 30 June 2023) [24]. NA: not applicable.

	Number of Studies	OR (95% CI)	I^2^	Q Test Chi^2^	Tau^2^	LP+ in HCV Subjects	LP+ in Control Group
All studies	15	3.65 [2.14–6.24]	61%	35.73	0.47	214+/59,221	231+/233,335
Diagnosis							
Records	2	5.18 [1.04–25.80]	96%%	24.31	1.29	146+/57,713	192+/230,852
Laboratory	13	3.03 [1.94–4.74]	0%	11.44	-	68+/1508	39+/2483
Geographic region ^1^							
Americas Region	3	2.42 [1.91–3.07]	49%	3.93	-	112+/34,464	185+/137,809
European Region	7	2.08 [0.95–4.52]	10%	6.67	-	31+/1014	7+/570
Eastern Mediterranean	1	5.51 [1.40–15.57]	NA	NA	-	14+/75	3+/75
Western Pacific	4	4.79 [1.93–11.86]	69%	9.54	0.57	57+/23,668	36+/94,881
Publication date							
1991–2000	4	3.68 [1.61–8.42]	0%	0.36	-	30+/729	14+/891
2001–2010	10	2.42 [1.95–3.02]	24%	11.88	-	142+/34,983	203+/138,408
≥2011	1	12.02 [6.56–22.01]	14	1.17		42+/23,509	14+/94,036
Risk of bias							
Low	11	4.16 [2.11–8.23]	66%	29.32	0.58	199+/58,967	205+/232,390
Medium	4	1.91 [1.00–3.64]	51%	6.15	-	15+/254	26+/945
Matched							
Yes gender & age	7	3.41 [1.28–9.08]	81%	31.95	1.00	169+/58,174	201+/231,318
Yes age	1	3.45 [0.45–26.22]	NA	NA	-	17+/505	1+/100
Not age & age	7	3.46 [1.97–6.11]	0%	3.28	0	28+/542	29+/11,917
Control group							
Health	7	2.97 [1.63–5.44]	0%	2.63	-	39+/908	15+/1195
Same Hospital	5	2.91 [0.83–10.21]	54%	8.68	1.02	26+/530	14+/1218
Records	2	5.18 [1.04–25.80]	96%	24.31	1.29	146+/57,713	192+/230,852
Volunteers	1	7.31 [0.37–144.22]	NA	NA	-	3+/70	0+/70
Time period							
Retrospective	2	5.18 [1.04–25.80]	96%	24.31	1.29	146+/57,713	192+/230,852
Prospective	13	3.03 [1.94–4.74]	0%	11.44	-	68+/1508	53+/2483
Sample size							
<100	7	4.32 [1.90–5.79]	0	3.18	-	35+/377	27+/1169
≥100	8	4.84 [1.98–11.78]	78%	27.76	0.80	179+/58,749	200+/232,166

**Table 3:** Pooled OR and 95% Confidence Interval (CI) of Hepatitis C in Lichen planus. Distribution by countries and decades. NA: Not applicable.

Country/Region	Number of Studies	OR [95% CI]	I^2^	Q Test Chi^2^	Tau^2^	Hepatitis+ in LP	Hepatitis + in Control Group
Arabia	4	7.56 [2.76–20.73]	0%	0.35	NA	41+/235	4+/187
1991–2000	1	5.34 [0.59–48.52]	NA	NA	NA	5+/34	1+/32
2001–2010	2	7.38 [2.15–25.29]	NA	NA	NA	30+/154	3+/105
≥2011	1	15.82 [0.87–289.12]	NA	NA	NA	6+/47	0+/50
Brazil	2	6.83 [3.35–13.93]					
2001–2010	2	6.83 [3.35–13.93]	44%	1.98	NA	11+/134	324+/45,673
China	3	0.92 [0.35–2.47]	0%	0.00	NA	7+/403	10/687
2001–2010	1	0.68 [0.19–2.46]	NA	NA	NA	4+/232	6+/240
≥2011	2	1.47 [0.31–6.93]	NA	NA	NA	3+/171	4+/447
Egypt	2	11.51 [0.41–322.53]	85%	6.81	4.95	30+/73	4+/60
1991–2000	1	2.38 [0.59–9.67]				9+/43	3+/30
2001–2010	1	67.67 [7.95–575.68]				21+/30	1+/30
Finland	1						
≥2011	1	NA				0+/152	0+/152
France	2	1.16 [0.47–2.87]					
1991–2000	2	1.16 [0.47–2.86]	0%	0.08		7+/154	17+/418
Germany	3	7.09 [2.11–23.85]	27%	1.37	NA	20+/492	3+/453
1991–2000	1	15.75 [2.01–123.31]				13+/84	1+/87
≥2011	2	3.46 [0.71–16.81]	NA	NA	NA	7+/408	2+/366
Hungary	1						
≥2011	1	5.65 [0.30–106.49	NA	NA	NA	4+/119	0+/72
India	9	6.70 [1.74–25.80]	0%			15+/551	1+/552
1991–2000	1	2.07 [0.10–44.50]	NA			2+/75	0+/30
2001–2010	2	7.34 [0.35–154.51]	NA			2+/144	0+/190
≥2011	6	9.04 [1.59–51,45]	0%	0.01	NA	11+/332	1+/332
Iraq	1						
≥2011	1	21.99 [4.38–110.41]				3+/97	3+/2070
Iran	9	4.25 [0.46–43.38]	88%	48.62	6.22	20+/1210	327+/320,649
2001–2010	5	5.59 [0.23–133.96]	91%	31.84	9.19	12+/405	323+/319,764
≥2011	4	2.02 [0.67–6.12]	0%	1.30	NA	8+/805	4+/885
Israel	2	4.33 [2.46–7.57]	0%	0.00	NA	33+/1619	79+/8567
2001–2010	1	4.21 [1.29–13.79]				3+/62	65+/5452
≥2011	1	4.35 [2.30–8.23]				30+/84	14+/3155
Italy	8 (11 cohorts)	3.92 [2.59–6.42]	59%	16.99	0.24	421+/22481	202+/2732
1991–2000	2	11.14 [4.68–26.62]	0%	0.28	NA	95+/333	6+/170
2001–2010	3	3.36 [1.81–6.26]	71%	6.99	0.21	305+/1241	188+/1953
≥2011	3	2.49 [1.09–5.68]	0%	0.88	NA	21+/674	8+/609
Japan	2	9.75 [5.02–19.72]	0%	0.09	NA	57+/104	17+/130
1991–2000	1	8.50 [2.28–31.73]				17+/45	3+/45
≥2011	1	10.68 [4.84–23.56				40+/59	14+/85
Nepal							
2001–2010	1	NA				0+/64	0+/43
Netherlands							
2001–2010	1	NA				0+/100	0+/100
New Zeland	1	0.72 [0.03–17.99]					
2001–2010	1	0.72 [0.03–17.99]				0+/77	1+/169
Nigeria	2	8.57 [1.54–47.76]	0%	0.01		18+/99	1+/54
2001–2010	1	9.60 [0.54–171.84]				9+/57	0+/24
≥2011	1	7.91 [0.94–66.25]				9+/42	1+/30
Pakistan	2	2.26 [0.20–25.94]	73%	3.71	2.36	19+/133	10+/188
2001–2010	1	6.20 [2.49–15.47]				18+/55	8+/110
≥2011	1	0.49 [0.04–5.56]				1+/78	2+/78
Poland	2						
≥2011	2	NA				0+/140	0+/186
Serbia	1						
2001–2010	1	NA				0+/48	0+/60
Slovenia	1	6.37 [0.30–133.36]					
2001–2010	1	6.37 [0.30–133.36]				2+/173	0+/218
Spain	4	6.48 [3.17–13.24]	0%			51+/479	9+/481
1991–2000	2	7.01 [3.04–16.17]	0%	0.42	NA	39+/178	7+/182
2001–2010	1	4.74 [1.00–22.54]	NA			9+/101	2+/99
≥2011	1	7.11 [0.36–138.47]	NA			3+/200	0/200
Taiwan	4	2.63 [1.33–5.21]	70%	9.90	1	89+/3011	447+/14,331
2001–2010	2	4.82 [0.68–34.13]	83%	5.79		37+/136	289+/1143
≥2011	2	2.32 [1.59–2.37]	75%	3.96		52+/2875	158+/13,188
Thailand	2	10.52 [1.93–57.34]	0%	0.01		14+/161	1+/161
2001–2010	1	11.99 [0.65–221.86]				5+/60	0+/60
≥2011	1	9.78 [1.22–78.72]				9+/101	1+/101
Turkey	6	4.09 [2.01–8.34]	0%	1.36	NA	29+/511	17+/970
1991–2000	2	5.29 [0.60–46.48]				5+/148	1+/148
2001–2010	4	3.58 [1.65–7.77]				24+/363	16+/822
UK	2	0.23 [0.01–5.85]					
1991–2000	2	0.23 [0.01–5.85]	NA			0+/100	1+/142
US	5	3.41 [1.74–5.59]	33%	5.94	NA	31+/535	17+/863
1991–2000	2	4.27 [1.72–10.62]	0%			19+/52	12+/81
2001–2010	1	3.80 [0.39–37.13]				4+/24	1+/20
≥2011	2	0.75 [0.16–3.39]				8+/459	4+/762
Multinational/multicenter ≥2011							
	1						
	1	9.65 [5.61–16.61]				47+/273	20+/948

**Table 4:** Pooled OR and 95 Confidence Interval (CI) of lichen planus in hepatitis C. Distribution by countries and decades. NA: Not applicable.

Country/Region	Number of Studies	OR [95% CI]	I^2^	Q Test Chi^2^	Tau^2^	LP in HCV+	LP in Control
Brazil	2	4.73 [1.55–14.42]					
2001–2010	2	4.73 [1.55–14.42]	35%	1.55		8/260	7/993
France							
1991–2000	1	0.96 [0.34–2.65]	38%	1.60		7/205	14/356
Japan	3	2.92 [1.46–5.85]	0%	0.87		15/159	22/845
1991–2000	2	3.61 [1.49–8.76]	0%	0.33		9/129	13/741
2001–2010	1	1.28 [0.54–3.04]	NA	NA		6/35	9/104
Kuwait							
2001–2010	1	5.51 [1.51–20.07]	NA			14/75	3/75
Poland	1						
2011–2021	1	2.47 [0.10–63.60]	NA	NA		1/23	0/29
Slovenia	1						
2001–2010	1	9.21 [0.49–172.49]	NA		NA	4/171	0/171
Spain	2	1.01 [0.33–3.14]	72%	3.56		17/600	5/200
1991–2000	1	3.45 [0.45–26.22]	NA			17/505	1/100
2001–2010	1	0.11 [0.01–2.11]	NA			0/95	4/100
Taiwan							
2011–2021	1	12.02 [6.56–22.01]				42/23,509	14/94,036
Turkey	2	2.25 [0.49–10.26]					
2001–2010	2	2.25 [0.49–10.26]	19%	NA	NA	5/120	2/120
US	1	2.34 [1.84–2.98]	NA				
2001–2010	1	2.34 [1.84–2.98]	NA			104/34,204	178/136,816

## Data Availability

The data presented in this study are available on request from the corresponding author.

## Supplementary Materials

The following supporting information can be downloaded at: https://www.mdpi.com/article/10.3390/jcm12185777/s1, Table S1: PRISMA item checklist; Appendix S1: Search terms in the electronic databases EMBASE, PubMed and WEB of SCIENCE; Table S2: General characteristics of the patients included in the study; Table S3: Pooled OR and 95% Confidence Interval (CI) of Hepatitis C in Lichen planus; Table S4: Pooled OR and 95% Confidence Interval (CI) of Hepatitis C in Lichen planus. Table S5: Pooled OR and 95% Confidence Interval (CI) of Hepatitis C in Lichen planus. Distribution by cities from Italy.
