# Supplementary material for: Bidirectional Association between Lichen Planus and Hepatitis C—An Update Systematic Review and Meta-Analysis
Source: J Clin Med. 2023 Sep 5;12(18):5777. doi: 10.3390/jcm12185777 (PMC10531646; doi:10.3390/jcm12185777)
Supplement: Supplementary file 1 [file jcm-12-05777-s001.zip › jcm-2572346-supplementary.pdf]

Online supplementary material

Supplemental S1. PRISMA item checklist

Table S1. PRISMA item checklist

Section and topic	Item #	Checklist item	Location where item is reported
**Title**			
Title	1	Identify the report as a systematic review.	1
**Abstract**			
Abstract	2	See the PRISMA 2020 for Abstracts checklist (table 2).	2,3
**Introduction**			
Rationale	3	Describe the rationale for the review in the context of existing knowledge.	4
Objectives	4	Provide an explicit statement of the objective(s) or question(s) the review addresses.	4
**Methods**			
Eligibility criteria	5	Specify the inclusion and exclusion criteria for the review and how studies were grouped for the syntheses.	5
Information sources	6	Specify all databases, registers, websites, organizations, reference lists and other sources searched or consulted to identify studies. Specify the date when each source was last searched or consulted.	Supplemental (S2) Appendix S1
Search strategy	7	Present the full search strategies for all databases, registers and websites, including any filters and limits used.	Supplemental (S2)
Selection process	8	Specify the methods used to decide whether a study met the inclusion criteria of the review, including how many reviewers screened each record and each report retrieved, whether they worked independently, and if applicable, details of automation tools used in the process.	Supplemental (S2)
Data collection process	9	Specify the methods used to collect data from reports, including how many reviewers collected data from each report, whether they worked independently, any processes for obtaining or confirming data from study investigators, and if applicable, details of automation tools used in the process.	Supplemental (S2)
Data items	10a	List and define all outcomes for which data were sought. Specify whether all results that were compatible with each outcome domain in each study were sought (e.g. for all measures, time points, analyses), and if not, the methods used to decide which results to collect.	Supplemental (S2)
	10b	List and define all other variables for which data were sought (e.g. participant and intervention characteristics, funding sources). Describe any assumptions made about any missing or unclear information.	Supplemental (S2)
Study risk of bias assessment	11	Specify the methods used to assess risk of bias in the included studies, including details of the tool(s) used, how many reviewers assessed each study and whether they worked independently, and if applicable, details of automation tools used in the process.	Supplemental (S2)
Effect measures	12	Specify for each outcome the effect measure(s) (e.g. risk ratio, mean difference) used in the synthesis or presentation of results.	Supplemental (S2)
Synthesis methods	13a	Describe the processes used to decide which studies were eligible for each synthesis (e.g. tabulating the study intervention characteristics and comparing against the planned groups for each synthesis (item #5)).	Supplemental (S2)
	13b	Describe any methods required to prepare the data for presentation or synthesis, such as handling of missing summary statistics, or data conversions.	Supplemental (S2)

Section and topic	Item #	Checklist item	Location where item is reported
	13c	Describe any methods used to tabulate or visually display results of individual studies and syntheses.	
	13d	Describe any methods used to synthesise results and provide a rationale for the choice(s). If meta-analysis was performed, describe the model(s), method(s) to identify the presence and extent of statistical heterogeneity, and software package(s) used.	Supplemental (S2)
	13e	Describe any methods used to explore possible causes of heterogeneity among study results (e.g. subgroup analysis, meta-regression).	Supplemental (S2)
	13f	Describe any sensitivity analyses conducted to assess robustness of the synthesised results.	Supplemental (S2)
Reporting bias assessment	14	Describe any methods used to assess risk of bias due to missing results in a synthesis (arising from reporting biases).	Supplemental (S2)
Certainty assessment	15	Describe any methods used to assess certainty (or confidence) in the body of evidence for an outcome.	Supplemental (S2)
**Results**			
Study selection	16a	Describe the results of the search and selection process, from the number of records identified in the search to the number of studies included in the review, ideally using a flow diagram (see fig 1).	5
	16b	Cite studies that might appear to meet the inclusion criteria, but which were excluded, and explain why they were excluded.	5, Figure 1
Study characteristics	17	Cite each included study and present its characteristics.	5-8
Risk of bias in studies	18	Present assessments of risk of bias for each included study.	8-9
Results of individual studies	19	For all outcomes, present, for each study: (a) summary statistics for each group (where appropriate) and (b) an effect estimate and its precision (e.g. confidence/credible interval), ideally using structured tables or plots.	6-7, table 1,2<br><br>Supplemental table
Results of syntheses	20a	For each synthesis, briefly summarise the characteristics and risk of bias among contributing studies.	6-7, table 1,2
	20b	Present results of all statistical syntheses conducted. If meta-analysis was done, present for each the summary estimate and its precision (e.g. confidence/credible interval) and measures of statistical heterogeneity. If comparing groups, describe the direction of the effect.	6-9, table 1,2
	20c	Present results of all investigations of possible causes of heterogeneity among study results.	table 1,2
	20d	Present results of all sensitivity analyses conducted to assess the robustness of the synthesised results.	Supplemental table S3, S4, Supplemental table S5
Reporting biases	21	Present assessments of risk of bias due to missing results (arising from reporting biases) for each synthesis assessed.	Supplemental table S3, S4, Supplemental table S5
Certainty of evidence	22	Present assessments of certainty (or confidence) in the body of evidence for each outcome assessed.	Supplemental table S3, S4, Supplemental table S5
**Discussion**			
Discussion	23a	Provide a general interpretation of the results in the context of other evidence.	9-13
	23b	Discuss any limitations of the evidence included in the review.	9-13
	23c	Discuss any limitations of the review processes used.	14
	23d	Discuss implications of the results for practice, policy, and future research.	14

Section and topic	Item #	Checklist item	Location where item is reported
**Other information**			
Registration and protocol	24a	Provide registration information for the review, including register name and registration number, or state that the review was not registered.	5
	24b	Indicate where the review protocol can be accessed, or state that a protocol was not prepared.	5
	24c	Describe and explain any amendments to information provided at registration or in the protocol.	
Support	25	Describe sources of financial or non-financial support for the review, and the role of the funders or sponsors in the review.	16
Competing interests	26	Declare any competing interests of review authors.	16
Availability of data, code, and other materials	27	Report which of the following are publicly available and where they can be found: template data collection forms; data extracted from included studies; data used for all analyses; analytic code; any other materials used in the review.	

Appendix S1. Search terms in the electronic databases EMBASE, PubMed and WEB of SCIENCE.

EMBASE

'lichen planus hepatitis' OR (('lichen'/exp OR lichen) AND planus AND ('hepatitis'/exp OR hepatitis)) OR 'oral lichen planus hepatitis c' OR (oral AND ('lichen'/exp OR lichen) AND planus AND ('hepatitis'/exp OR hepatitis) AND c) OR 'lichen hepatitis' OR (('lichen'/exp OR lichen) AND ('hepatitis'/exp OR hepatitis)) OR 'lichen hepatitis c' OR (('lichen'/exp OR lichen) AND ('hepatitis'/exp OR hepatitis) AND c) OR 'oral lichen planus hepatitis' OR (oral AND ('lichen'/exp OR lichen) AND planus AND ('hepatitis'/exp OR hepatitis)) OR 'lichen planus hepatitis c' OR (('lichen'/exp OR lichen) AND planus AND ('hepatitis'/exp OR hepatitis) AND c) OR 'hepacivirus lichen' OR (('hepacivirus'/exp OR hepacivirus) AND ('lichen'/exp OR lichen)) OR 'hepacivirus oral lichen planus' OR (('hepacivirus'/exp OR hepacivirus) AND oral AND ('lichen'/exp OR lichen) AND planus) OR 'infection lichen' OR (('infection'/exp OR infection) AND ('lichen'/exp OR lichen)) OR 'infection oral lichen planus' OR (('infection'/exp OR infection) AND oral AND ('lichen'/exp OR lichen) AND planus) OR 'oral lichen planus liver disease' OR (oral AND ('lichen'/exp OR lichen) AND planus AND ('liver'/exp OR liver) AND ('disease'/exp OR disease)) OR 'lichen planus liver disease' OR (('lichen'/exp OR lichen) AND planus AND ('liver'/exp OR liver) AND ('disease'/exp OR disease)) OR 'hepatitis c oral lichen planus' OR (('hepatitis'/exp OR hepatitis) AND c AND oral AND ('lichen'/exp OR lichen) AND planus) OR 'hepatitis c lichen planus' OR (('hepatitis'/exp OR hepatitis) AND c AND ('lichen'/exp OR lichen) AND planus) OR 'hepatitis oral lichen planus' OR (('hepatitis'/exp OR hepatitis) AND oral AND ('lichen'/exp OR lichen) AND planus) OR 'hepatitis lichen planus' OR (('hepatitis'/exp OR hepatitis) AND ('lichen'/exp OR lichen) AND planus) OR 'transaminase oral lichen planus' OR (('transaminase'/exp OR transaminase) AND oral AND ('lichen'/exp OR lichen) AND planus) OR 'transaminase lichen planus' OR (('transaminase'/exp OR transaminase) AND ('lichen'/exp OR lichen) AND planus) OR 'liver disease oral lichen planus' OR (('liver'/exp OR liver) AND ('disease'/exp OR disease) AND oral AND ('lichen'/exp OR lichen) AND planus) OR 'liver disease lichen planus' OR (('liver'/exp OR liver) AND ('disease'/exp OR disease) AND ('lichen'/exp OR lichen) AND planus) OR 'vaginal lichen planus hepatitis' OR (vaginal AND ('lichen'/exp OR lichen) AND planus AND ('hepatitis'/exp OR hepatitis)) OR 'vaginal lichen planus liver disease' OR (vaginal AND ('lichen'/exp OR lichen) AND planus AND ('liver'/exp OR liver) AND ('disease'/exp OR disease)) OR 'lichen planopilaris liver disease' OR (('lichen'/exp OR lichen) AND planopilaris AND ('liver'/exp OR liver) AND ('disease'/exp OR disease)) OR 'lichen planus pilaris liver disease' OR (('lichen'/exp OR lichen) AND planus AND pilaris AND ('liver'/exp OR liver) AND ('disease'/exp OR disease)) OR 'genital lichen planus' OR (genital AND ('lichen'/exp OR lichen) AND planus) OR 'glande lichen planus' OR (glande AND ('lichen'/exp OR lichen) AND planus) OR 'vulvar lichen planus hepatitis' OR (vulvar AND ('lichen'/exp OR lichen) AND planus AND ('hepatitis'/exp OR hepatitis)) OR 'sclerosus lichen planus hepatitis' OR (sclerosus AND ('lichen'/exp OR lichen) AND planus AND ('hepatitis'/exp OR hepatitis)) OR 'nail lichen' OR (('nail'/exp OR nail) AND ('lichen'/exp OR lichen)) OR 'cutaneous lichen hepatitis' OR (cutaneous AND ('lichen'/exp OR lichen) AND ('hepatitis'/exp OR hepatitis)) OR 'cutaneous lichen hepatitis c' OR (cutaneous AND ('lichen'/exp OR lichen) AND ('hepatitis'/exp OR hepatitis) AND c) OR 'cutaneous lichen liver disease' OR (cutaneous AND ('lichen'/exp OR lichen) AND ('liver'/exp OR liver) AND ('disease'/exp OR disease)) OR 'cutaneous lichen transaminase' OR (cutaneous AND ('lichen'/exp OR lichen) AND ('transaminase'/exp OR transaminase))

PubMed

(((Lichen planus hepatitis) OR (Oral lichen planus hepatitis)) OR (Lichen planus hepatitis C)) OR (Oral lichen planus hepatitis C)) OR (lichen hepatitis)) OR (lichen hepatitis C)) OR (hepacivirus lichen)) OR (hepacivirus oral lichen planus)) OR (infection lichen)) OR (infection oral lichen planus) OR (ORAL LICHEN PLANUS liver disease)) OR (lichen planus liver disease) OR (Hepatitis C oral lichen planus) OR (Hepatitis C lichen planus)) OR (hepatitis oral lichen planus)) OR (hepatitis lichen planus sport by: Most Recent) OR (transaminase oral lichen planus)) OR (transaminase lichen planus)) OR (liver disease oral lichen planus)) OR (liver disease lichen planus)) OR (vaginal lichen planus hepatitis)) OR (vaginal lichen planus liver disease)) OR (lichen planopilaris liver disease)) OR (lichen planus pilaris liver disease)) OR (genital lichen planus OR glande lichen planus)) OR (sclerosus lichen planus hepatitis)) OR (nail lichen)) OR (cutaneous lichen hepatitits OR cutaneous lichen hepatitis C OR cutaneous lichen liver disease)) OR OR cutaneous transaminase)

WEB of SCIENCE

Lichen planus hepatitis (Topic) or Oral lichen planus hepatitis (Topic) or cutaneous lichen liver disease (Topic) or Lichen planus hepatitis C (Topic) or Oral lichen planus hepatitis C (Topic) or lichen hepatitis (Topic) or lichen hepatitis C (Topic) or hepacivirus lichen (Topic) or hepacivirus oral lichen planus (Topic) or infection lichen (Topic) or infection oral lichen planus (Topic) or oral lichen planus liver disease (Topic) or lichen planus liver disease (Topic) or Hepatitis C oral lichen planus (Topic) or Hepatitis C lichen planus (Topic) or hepatitis oral lichen planus (Topic) or hepatitis lichen planus (Topic) or transaminase oral lichen planus (Topic) or transaminase lichen planus (Topic) or liver disease oral lichen planus (Topic) or liver disease lichen planus (Topic) or vaginal lichen planus hepatitis (Topic) or vaginal lichen planus liver disease (Topic) or lichen planopilaris liver disease (Topic) or lichen planus pilaris liver disease (Topic) or genital lichen planus (Topic) or glands lichen planus (Topic) or sclerosus lichen planus hepatitis (Topic) or nail lichen (Topic) or cutaneous lichen hepatitis (Topic) or cutaneous lichen hepatitis C (Topic) or cutaneous transaminase (Topic)

Subjects	Number of studies	HCV+ in LP patients		LP in HCV+ Subjects
**Mean age**		49.82 (48.36-51.27)		46.96 (43.08-50.85)
**Child lichen**	1	0/30		
**Female/male**		1.70/1		1/3.18
**Female genital**	2	0/120		
**Male genital**	2	1/150		
**Lichen planopilaris**	3	3/342		
**Qualitative and quantitative studies**	192	143		49
**Location by WHO**		Global Prevalence % (CI: confidence interval)		Global Prevalence % (CI: confidence interval)
Africa	2	18.56 (-17.77 -54.89)	1	23.48%
America	14	11.54 (1.8-21.25)	4	2.90 (0.68-5.11)
Eastern Mediterranean	26	9.61 (3.55-15.68)	13	11.10 (5.29-16.91)
Europe	69	7.99 (5.87-10.12)	22	3.72 (1.87-5.57)
South East Asia	16	3.43 (0.41-6.45)	1	0
Western Pacific	16	19.18 (6.32-32,03)	8	10.55 (4.18-16.92)
**Country**				
Brazil	6	3.90 [0.26-7.55]	4	2.90 (0.68-5.11)
Bulgaria			1	5.88%
China	4	3.60 [-2.79-9.49]	1	0.67%
Croatia	1	3.12%		
Egypt	3	36.02 [-37.26-109.26]	1	3.87%
Finland	1	0		
France	4	3.29 [-2.30-7.59]	4	3.23 (0.55-5.90)
Germany	6	3.87 [-2.32-10.57]	3	2.21 (-3.10-7.52
Hungary	1	3,36%		
India	13	2.90 [0.63-6.44]	1	0
Iran	10	1.57 [0.35-2.80]		
Iraq	1	3.09%	1	1.36%
Israel	2	3.37 [-15-11-21.86]		
Italy	24	12.08 [7.96-16.17]	4	2.15 (0.05-4.25)
Japan	5	40.54 [3.95-77.13]		
Kuwait	1	0	1	18.66%
Morocco			1	23.48%
Nepal	1	0		
Netherlands	2	0		
New Zeland	1	0		

Nigeria	2	18.56 [-17.77-54.89]		
Oman	1	2,83		
Pakistan	7	12.89 [2.28-23.49]	10	16.95 (3.91-29.99)
Poland	2	0	2	2.75 (-17.38-22.89)
Romania	1	13.37%		
Saudi Arabia	4	7.52 [-4.07-19.12]		
Serbia	1	0		
Slovenia	1	1.15%	1	2.33%
Spain	10	10.38 [5.19-15.56]	2	1.68 (-19.66-23.02)
Sweeden	1	0,83%		
Switherland	1	0,68%		
Taiwan	5	17.47 [-8.85-39.80]	1	0.17%
Thailand	2	8.62 [4.93-12.30]		
Turkey	6	5.98 [1.59-10.37]	3	5.51 (0.39-11.41)
UK	4	0	1	20%
US	8	16.53 [0.64-33.69]	1	0.3%
Multinational/multicenter	1	17.21%		

Table S2. General characteristics of the patients included in the study. Demographic and geographic data.

Author/year	Events[1]	Total[1]	Events[2]	Total[2]	Weight	OR (95%CI)
Abdel Haq	0	56	0	56		Not estimable
Adamo, 2022	10	300	4	300	2.0%	2.55 [0.79, 8.23]
Ali, 2007	0	40	0	40		Not estimable
Amer, 2007	21	30	1	30	1.1%	67.67 [7.95, 575.68]
Arduino, 2017	8	307	2	242	1.7%	3.21 [0.68, 15.26]
Asaad & Samdani, 2005	30	114	3	65	2.1%	7.38 [2.15, 25.29]
Bagán, 1998	23	100	5	100	2.5%	5.68 [2.06, 15.62]
Beaird, 2001	4	24	1	20	1.0%	3.80 [0.39, 37.13]
Bellman, 1995	7	30	2	41	1.6%	5.93 [1.14, 31.02]
Birkenfeld, 2011	30	1557	14	3115	3.2%	4.35 [2.30, 8.23]
Bokor-Bratic, 2004	0	48	0	60		Not estimable
Campisi, 2004	238	859	154	1209	3.7%	2.63 [2.10, 3.29]
Carrozzo PCR, 1996	15	70	3	70		Not estimable
Chuang, 1999	12	22	10	40	2.3%	3.60 [1.19, 10.85]
Chung, 2004	14	32	287	1043	3.0%	2.05 [1.01, 4.17]
Colquhoun, 2004	0	77	1	169	0.5%	0.72 [0.03, 17.99]
Cribier, 1994	2	52	3	112	1.4%	1.45 [0.24, 8.97]
Daramola, 2002 a	9	57	0	24	0.7%	9.60 [0.54, 171.84]
Das, 2006	2	104	0	150	0.6%	7.34 [0.35, 154.51]
Dave, 2020	3	156	4	156	1.7%	0.75 [0.16, 3.39]
Denli, 2004	7	140	4	280	2.1%	3.63 [1.04, 12.62]
Ding, 2017	2	21	0	18	0.6%	4.74 [0.21, 105.54]
Dupin, 1997	5	102	14	306	2.4%	1.08 [0.38, 3.06]
El-Rifaei, 1999	5	34	1	32	1.1%	5.34 [0.59, 48.52]
Erkek, 2001	7	54	2	54	1.6%	3.87 [0.77, 19.57]
Figueiredo, 2002	6	68	14	726	2.5%	4.92 [1.83, 13.26]
Garg, 2002	0	64	0	43		Not estimable
Gerayli, 2015	3	134	0	134	0.7%	7.16 [0.37, 139.96]
Ghaderi & Makhmalbaf, 2007	3	73	1	150	1.0%	6.39 [0.65, 62.49]
Ghodsi, 2004	7	146	319	319375	2.9%	50.37 [23.39, 108.47]
Giménez-García, 2003	9	101	2	99	1.7%	4.74 [1.00, 22.54]
Giuliani 2007	9	79	25	466	2.9%	2.27 [1.02, 5.06]
Guerreiro, 2005	5	66	310	44947	2.6%	11.80 [4.71, 29.57]
Halawani, 2014	6	47	0	50	0.7%	15.82 [0.87, 289.12]
Harman, 2004	8	128	1	128	1.1%	8.47 [1.04, 68.71]
Ibrahim, 1999	9	43	3	30	1.9%	2.38 [0.59, 9.67]
Ilter, 1998	0	75	0	75		Not estimable
Imhof, 1997	13	84	1	87	1.2%	15.75 [2.01, 123.31]
Ingafou, 1998	0	55	0	110		Not estimable
Jayavelu & Sambandan, 2012	0	30	0	30		Not estimable

Karavelioğlu, 2004	2	41	9	360	1.7%	2.00 [0.42, 9.59]
Kirtak, 2000	5	73	1	73	1.1%	5.29 [0.60, 46.48]
Klanrit, 2003	5	60	0	60	0.7%	11.99 [0.65, 221.86]
Konidena & Pavani, 2011	3	25	0	25	0.6%	7.93 [0.39, 162.07]
Kumar, 2019	0	75	0	75		Not estimable
Laeijendecker, 2005	0	100	0	100		Not estimable
Laniosz, 2019	5	303	0	606	0.6%	22.35 [1.23-405-53]
Lin, 2010	23	104	2	100	1.8%	13.91 [3.18, 60.79]
Lodi, 2004	58	303	9	278	3.0%	7.08 [3.43, 14.58]
López-Jornet, 2012	3	200	0	200	0.6%	7.11 [0.36, 138.47]
Ma, 2022	8	421	129	8420	2.7%	1.24 [0.61, 2.56]
Manomaivat, 2018	9	101	1	101	1.1%	9.78 [1.22, 78.72]
Mignona, 1998	76	263	3	100	2.2%	13.14 [4.04, 42.74]
Mogaddam & Anamzade, 2010	1	60	0	60	0.6%	3.05 [0.12, 76.39]
Nagao & Sata, 2012	40	59	14	85	2.9%	10.68 [4.84, 23.56]
Narayan, 1998	2	75	0	30	0.6%	2.07 [0.10, 44.50]
Nasimi, 2022	1	208	0	208	0.5%	2.10 [0.09, 51.86]
Noreen, 2018	1	78	2	78	0.9%	0.49 [0.04, 5.56]
Nosratzahi, 2018	0	50	0	50		Not estimable
Patil, 2012	0	130	0	130		Not estimable
Petti, 2011	4	413	4	493	1.9%	1.20 [0.30, 4.81]
Pippi, 2016	3	67	2	67	1.2%	1.52 [0.25, 9.42]
Pitak-Arnnop, 2021	47	273	20	948	3.3%	9.65 [5.61, 16.61]
Rahnama, 2005	1	66	3	140	1.0%	0.70 [0.07, 6.88]
Remmerbach, 2016	0	143	0	109		Not estimable
Rübsam, 2011	7	265	2	257	1.6%	3.46 [0.71, 16.81]
Siponen, 2010	0	152	0	152		Not estimable
Sobti, 2016	0	30	0	30		Not estimable
Song, 2016	1	150	4	429	1.1%	0.71 [0.08, 6.43]
Sreedevi, 2016	8	42	1	42	1.1%	9.65 [1.15, 81.02]
Stojanovic, 2008a	2	71	0	218	0.6%	6.37 [0.30, 133.56]
Strak, 2014	3	97	3	2070	1.6%	21.99 [4.38, 110.41]
Szarka, 2009	4	119	0	72	0.6%	5.65 [0.30, 106.49]
Sánchez-Pérez, 1996	16	78	2	82	1.7%	10.32 [2.29, 46.58]
Taghavi Zenouz, 2010	0	60	0	39		Not estimable
Tameez-ud-deen, 2003	18	55	8	110	2.6%	6.20 [2.49, 15.47]
Tanei, 1995	17	45	3	45	2.0%	8.50 [2.28, 31.73]
Tucker, 1999	0	45	1	32	0.6%	0.23 [0.01, 5.85]
Udayashankar, 2008	0	40	0	40		Not estimable
Ukonu & Uhummwangho, 2012	9	42	1	30	1.1%	7.91 [0.94, 66.25]
Wang, 2020	44	2454	29	4768	3.4%	2.98 [1.86, 4.78]
Yarom, 2007	3	62	65	5452	2.2%	4.21 [1.29, 13.79]
Zhou, 2010	4	232	6	240	2.0%	0.68 [0.19, 2.46]

Zychowska & Zychowska, 2020	0	84	0	130	Not estimable
Total events	969	13495	1515	401386	4.48 [3.48-5.77]

Heterogeneity: $Tau^2 = 0.47$; $Chi^2 = 165.42$, df = 66 ($P < 0.00001$); $I^2 = 60\%$

Test for overall effect: $Z = 11.66$ ($P < 0.00001$)

Table S3. Pooled OR and 95% Confidence Interval (CI) of Hepatitis C in Lichen planus. 1 Experimental group. 2. Control group.

Table S4. Pooled OR and 95% Confidence Interval (CI) of Lichen planus in hepatitis C patients. 1 Experimental group. 2. Control group.

Author/year	Events[1]	Total[1]	Events[2]	Total[2]	Weight	OR (95%CI)
Al-Ali 2011	14	75	3	75	8.3%	5.51 [1.51, 20.07]
Bagán, 1998	17	505	1	100	4.9%	3.45 [0.45, 26.22]
Cribier, 1998	4	100	0	50	2.7%	4.71 [0.25, 89.22]
Cunha, 2005	2	134	1	95	3.8%	1.42 [0.13, 15.94]
Dervis, 2005	3	70	0	70	2.7%	7.31 [0.37, 144.22]
El-Serag, 2002	104	34204	178	136816	15.6%	2.34 [1.84, 2.98]
Figueiredo, 2002	6	126	6	898	9.2%	7.43 [2.36, 23.42]
Ma, 2021	42	23509	14	94036	13.3%	12.02 [6.56, 22.01]
Maticic, 2008	4	171	0	171	2.8%	9.21 [0.49, 172.49]
Micó, 2004	0	95	4	100	2.8%	0.11 [0.01, 2.11]
Nagao, 1997	4	84	6	591	8.3%	4.88 [1.35, 17.65]
Nagao, 2000	5	40	7	150	8.9%	2.92 [0.87, 9.74]
Nagao, 2005	6	35	9	104	9.5%	2.18 [0.72, 6.65]
Soylu, 2007	2	50	2	50	5.0%	1.00 [0.14, 7.39]
Sulka, 2004	1	23	0	29	2.3%	3.93 [0.15, 101.17]
Total (95% CI)	214	59221	231	233335	100.0%	3.65 [2.14, 6.24]

Naples & Bari [75]	13.14 [4.04-18.70]
Brescia (2004) [10]	10.59 [3.00-37.35]
Campania (2004) [33]	1.91 [1.43-2.57]
Foggia (2007) [55]	2.27 [1.02-5.06]
Milan (2004) [10]	6.05 [2.06-17.80]
Rome (2004) [10]	4.93 [1.03-23.58]
Sicilia (2004)[33]	3.84 [2.54-5.80]
Piemonte	
1996 (March 1992-July 1994) [34]	8.32 [2.33-29.66]
2017 (January 2015- May 2017) [26]	3.21 [0.68-15.26]

Table S5. Pooled OR and 95% Confidence Interval (CI) of Hepatitis C in Lichen planus. Distribution by cities from Italiy.
